# Fermenting kale (*Brassica oleracea* L.) enhances its functional food properties by increasing accessibility of key phytochemicals and reducing antinutritional factors

**DOI:** 10.1002/fsn3.4195

**Published:** 2024-05-06

**Authors:** Ujjwol Subedi, Samnhita Raychaudhuri, Si Fan, Opeyemi Ogedengbe, Diana N. Obanda

**Affiliations:** ^1^ Department of Nutrition and Food Sciences University of Maryland College Park Maryland USA

**Keywords:** anti‐inflammatory, antioxidant, fermentation, kale, polyphenols, sulforaphane

## Abstract

The properties of kale as a functional food are well established. We sought to determine how fermentation further enhances these properties. We tested different fermentation conditions: (i) spontaneous fermentation with naturally occurring bacteria, (ii) spontaneous fermentation with 2% salt, (iii) *Lactococcus lactis*, (iv) *Lactobacillus acidophilus*, (v) mixture of *L. lactis* and *L. acidophilus*, (vi) mixture of *L. lactis*, *L. acidophilus*, and *Clostridium butyricum.* We quantified selected bioactive components using high‐performance liquid chromatography (HPLC) and antinutritional factors using a gravimetric method and spectrophotometry. We then determined (i) the antioxidant capacity of the vegetable, (ii) anti‐inflammation capacity, and (iii) the surface microbiota composition by 16S sequencing. All fermentation methods imparted some benefits. However, fermentation with mixed culture of *L. lactis* and *L. acidophilus* was most effective in increasing polyphenols and sulforaphane accessibility, increasing antioxidant activity, and reducing antinutritional factors. Specifically, fermentation with *L. lactis and L. acidophilus* increased total polyphenols from 8.5 to 10.7 mgGAE/g (milligrams of gallium acid equivalent per gram) and sulforaphane from 960.8 to 1777 μg/g (microgram per gram) but decreased the antinutritional factors oxalate and tannin. Total oxalate was reduced by 49%, while tannin was reduced by 55%–65%. The antioxidant capacity was enhanced but not the anti‐inflammation potential. Both unfermented and fermented kale protected equally against lipopolysaccharide (LPS)‐induced inflammation in RAW 264.7 macrophages and prevented increases in inducible nitric oxide synthase (iNOS), tumor necrosis factor‐alpha (TNF‐α), interleukin‐1 beta (IL‐1β), and interleukin‐6 messenger RNA (IL‐6 mRNA) expression by 84.3%, 62%, 68%, and 85.5%, respectively. Unfermented and naturally fermented kale had high proportions of sulfur reducing Desulfubrio and Proteobacteria usually associated with inflammation. Fermenting with *L. lactis* and/or *L. acidophilus* changed the bacterial proportions, reducing the Proteobacteria while increasing the genera *Lactobacilli* and *Lactococcus.* In summary, fermentation enhances the well‐known beneficial impacts of kale. Fermentation with mixed cultures of *L. lactis* and *L. acidophilus* imparts higher benefits compared to the single cultures or fermentation with native bacteria present in the vegetable.

## Graphical abstract

All fermentation methods tested imparted some benefits. However, fermentation with mixed culture of *Lactococcus lactis* and *Lactobacillus acidophilus* was most effective in increasing polyphenols and sulforaphane accessibility, increasing antioxidant activity, and reducing antinutritional factors. Both fermented and unfermented kale exhibit strong anti‐inflammatory effects.

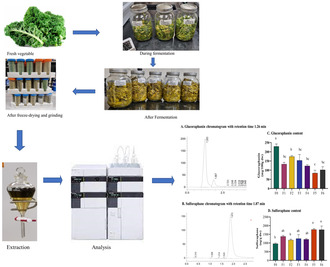

## 1 INTRODUCTION

Green curly kale (*Brassica oleracea* L. convar. acephala var. sabellica) is a leafy vegetable recognized by ruffled leaves, fibrous stalks, and deep green color, and has been a food crop since 2000 B.C. in the eastern Mediterranean and Asia regions (Šamec et al., 2018). Although the cultivation and consumption of kale have spread worldwide through immigrants, travelers, and merchants, the most popular cruciferous vegetable in the United States is broccoli. Its phytochemistry and biological activity have been most extensively studied and reviewed compared to those of other cruciferous vegetables. However, in the last couple of years, the popularity of kale has increased. There is even an event celebrated in the United States as National Kale Day (the first Wednesday in October). The celebration casts light on kale's versatility, amazing health benefits, and increases awareness for its cultivation and inclusion in diets (Šamec et al., 2018). Generally, kale is consumed as a raw salad or beverage, but its use in novel bioactive food formulations is gaining interest (Michalak et al., 2020).

Listed as one of the powerhouse fruits and vegetables (PFV), the nutraceutical industry has recently taken notice of kale's health benefits (Khalid et al., 2023; Nayak et al., 2015; Thavarajah et al., 2016), as it is rich in flavonoids, polyphenols, carotenoids, glucosinolates, and dietary soluble and insoluble fiber (Michalak et al., 2020). Kale is also rich in Na, K Mg, Ca, and Fe and stands out among other cruciferous vegetables as the best source of folate, nicotinic acid (vitamin B3), and vitamins C, K, A, B6, B2, and B1. Compared to other cruciferous vegetables including broccoli, cabbage, spinach, and Brussels sprouts, kale contains higher amounts of beta‐carotene, lutein, zeaxanthin, glucosinolates, and sulforaphane (Aǧagündüz et al., 2022). Kale contains 7–10 times the amount of sulforaphane in broccoli; kale contains 1736–3027 μg/g sulforaphane compared to the 260 μg/g sulforaphane in broccoli. Sulforaphane is an isothiocyanate organosulfur compound shown to have a myriad of anti‐inflammation properties (Aǧagündüz et al., 2022).

Fermented foods like kefir, kombucha, sauerkraut, tempeh, natto, miso, kimchi, and sourdough bread are produced through microbial growth and bacterial enzymatic action on food components. They are becoming popular globally because of health benefits from the probiotic effect of their constituent microorganisms, fermentation‐derived bioactive peptides, conversion of phenolic compounds into biologically active compounds, as well as the reduction of antinutritional factors (Dimidi et al., 2019). Notable antinutritional factors in foods, especially green vegetables, include nitrates, phytates, tannins, oxalates, and cyanogenic glycosides that interfere with nutrient absorption and have been associated with various health risks. Lactic acid fermentation based on the production of lactic acid from sugar helps to prevent spoilage and enriches nutrients and sensory attributes in the food (Šalić & Šamec, 2022). Generally, the lactic acid fermentation process can be done spontaneously by indigenous microbial cultures on vegetables (traditional method) or by adding selected starter cultures (Li & Gänzle, 2020). Different brassica vegetables are consumed as fermented vegetables. However, few studies have focused on the fermentation of kale and how it may impact its functional food properties.

Recent studies have shown that kale improves metabolic health, attenuates inflammation, and modulates gut microbial composition (Raychaudhuri et al., 2021; Shahinozzaman et al., 2021). Herein, we sought to determine how fermentation further improves the functional food properties of kale. Our objectives were to determine the impact of fermentation on (i) the concentration or accessibility of selected bioactive components and antinutritional factors of kale, (ii) the antioxidant and anti‐inflammatory capacity of kale, and (iii) the surface microbiota composition. We hypothesized that fermentation improves the bioavailability or accessibility of bioactive components and reduces the amount of antinutritional factors. We compared six different fermentation methods that included traditional practices and inoculation with different bacterial species. We report on the impact of the different fermentation methods on the variables tested.

## 2 MATERIALS AND METHODS

### 2.1 Kale vegetable sample

The sample vegetable was purchased in one batch at the Farmer's Market in Downtown Silver Spring, Maryland. It was all from one farm (Spring Valley Farm in Romney, West Virginia). According to the details provided by the farm, the variety of the kale was Winter bor.

### 2.2 Kale fermentation

Fresh kale leaves were cleaned, chopped into small pieces (~2–3 cm), mixed up, and divided into seven groups of 1200 g each. Each group was further divided into three sections of 400 g to give three replicates for each treatment. The seven treatment groups are shown in Table 1. Two groups followed traditional household methods; spontaneous fermentation with naturally occurring bacteria and fermentation with 2% salt (NaCl) based on vegetable weight. The other four groups were inoculated with different bacterial species combinations, as shown in Table 1. The three bacterial taxa were selected based on literature supporting their role as probiotics and availability to the research group. The selection of bacterial species was not based on use as starter culture in any previous studies.

**TABLE 1:** Fermentation treatments were used in the study.

Sample groups	
F0	Unfermented control
F1	Spontaneous fermentation with naturally occurring bacteria
F2	Fermentation with 2% salt (% based on vegetable weight)
F3	*Lactococcus lactis* (ATCC 11454)
F4	*Lactobacillus acidophilus* (ATCC 314)
F5	*Lactococcus lactis* and *Lactobacillus acidophilus* mixture
F6	*Lactococcus lactis*, *Lactobacillus acidophilus*, and *Clostridium butyricum* (ATCC 19398) mixture

**FIGURE 1:**
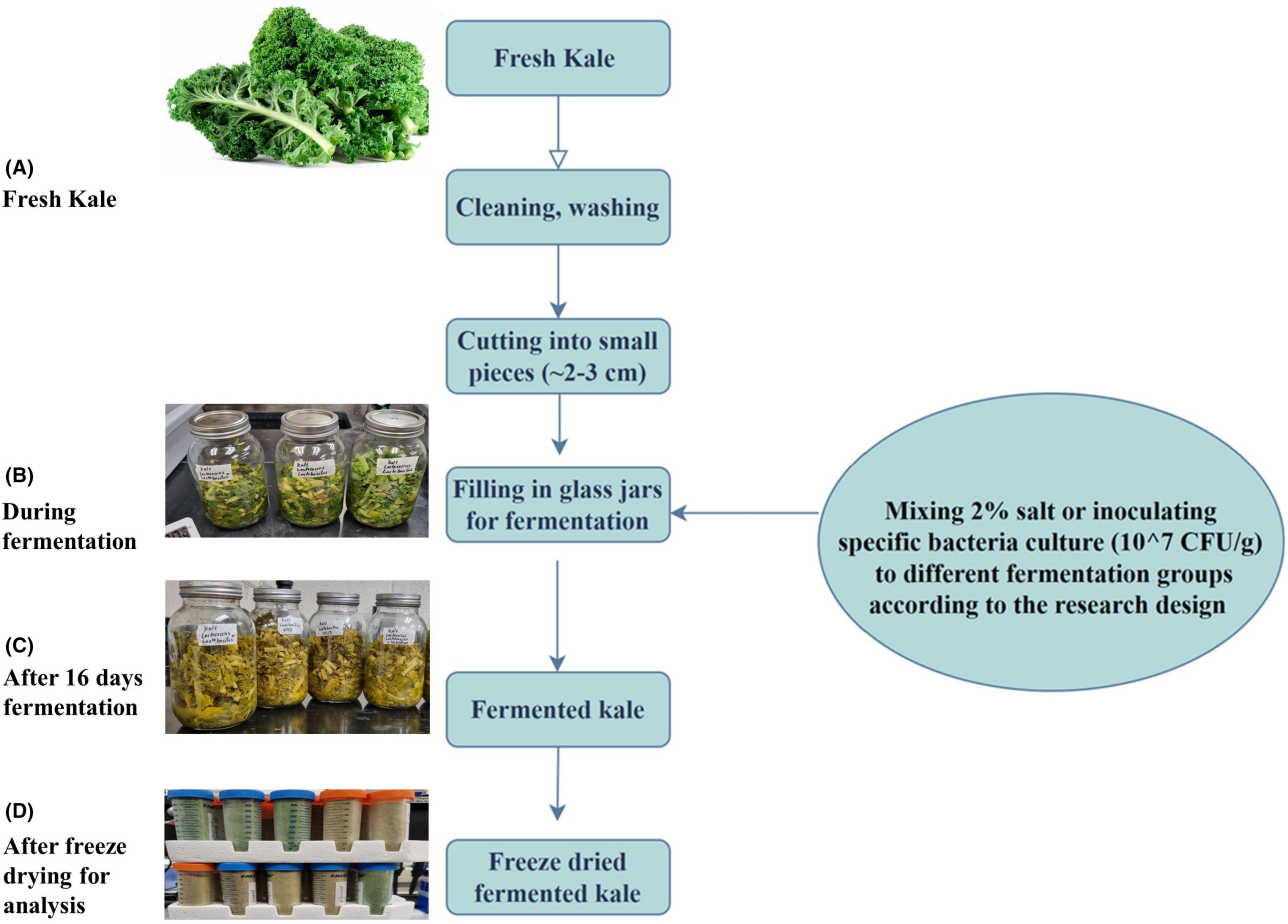
(A–D) Flowchart of kale fermentation and sample processing steps. Fresh kale leaves were cleaned, chopped, packed in jars, and fermented at room temperature for 16 days. After fermentation, samples (all vegetable plus the liquid in the jar) were freeze‐dried and then ground to a fine powder and stored in an airtight container at −20°C until further analysis. Unfermented fresh kale leaves were also chopped and immediately freeze‐dried and stored in an airtight container at −20°C until further analysis.

While fermentation was taking place in groups F1–F6, the unfermented control F0 was kept frozen at −20°C. For group F1, vegetable pieces were put into 1 liter mason jars, pressed with the sterile pestle, sealed, and left to ferment naturally. For group F2, 2% table salt w/w was added to the vegetable pieces and mixed to ensure equal distribution before placing them into the jars. For groups F3–F6, the specific bacterial cultures shown in Table 1 were added to the vegetable (10^7^ CFU/g ratio) and mixed before placing them in the jars, as shown by Ghimire et al. (2020) and Michalak et al. (2018). All fermentations were conducted at room temperature (20–25°C) for 16 days. All components of the individual jars (vegetable plus liquid) were freeze‐dried and used in subsequent tests (Figure 1).

### 2.3 Determination of pH and titratable acidity

After 16 days, the pH was determined using a digital pH meter, before each sample, including the liquid in the jars, was freeze‐dried and ground into a fine powder using a food grinder and stored in an airtight container at −20°C until further analysis. The fresh unfermented kale was crushed using a mortar and pestle and the pH was measured by inserting the pH probe in the crushed fresh vegetable before freeze‐drying and powdering. To determine percent acidity, the method used by Cai et al. (2019) was adopted. Briefly, 2.0 g of each powdered sample was homogenized, suspended in 10 mL deionized (DI) water, and titrated with 0.1N NaOH with phenolphthalein as indicator.

### 2.4 Determination of total phenolic content

Polyphenols were determined by the method described by Chin et al. (2022). Briefly, 2.0 g kale sample was blended in 20 mL of 80% (v/v) methanol, placed on a shaker plate for 30 min, vortexed for 1 min, and centrifuged at 4000 rpm (revolutions per minute) and 30°C for 10 min. The supernatant was filtered through Whatman No. 1 filter paper. The process was repeated, the supernatants were combined and adjusted to a final volume of 50 mL using 80% (v/v) methanol solvent. The filtrate was stored at −20°C until later use for the determination of polyphenol and flavonoid contents. The total polyphenol content (TPC) was measured using a modified Folin–Ciocalteu spectrophotometric method. Exactly 200 μL sample extract, 1.5 mL sodium carbonate (Na_2_CO_3_) (75 g/L), and 1.5 mL of a freshly diluted (10‐fold) Folin–Ciocalteu reagent were mixed and vortexed. The solution was incubated in the dark for 2 h at room temperature (RT). Absorbance was determined at 725 nm using a ultraviolet (UV) spectrophotometer (Thermo Scientific, Genesys 20). Gallic acid (GA) was used to prepare a standard curve ranging from 0.1 to 0.8 mg/mL. The TPC was expressed in milligrams of gallic acid equivalents (GAE) per 100 g sample on a dried weight basis (mg GAE/100 g db).

### 2.5 Determination of total flavonoid content

Total flavonoids were determined using the aluminum trichloride (AlCl_3_) colorimetric method, as outlined by Chang et al. (2020). Exactly 1 g of freeze‐dried powder sample was blended with 10 mL of 80% (v/v) ethanol, placed on a shaker plate for 30 min, vortexed for 1 min, and then centrifuged at 4000 rpm and 30°C for 10 min. The supernatant was filtered through a Whatman No. 1 filter paper. The extraction process was repeated on the residue and the supernatants were combined and adjusted to a final volume of 25 mL using 80% (v/v) ethanol. The filtrate was stored at −20°C for use for quantifying total flavonoid content. Quercetin was utilized as the standard; 10 mg quercetin was dissolved in 10 mL of 80% ethanol that was serially diluted to construct a standard curve ranging from 25 to 100 μg/mL. Exactly 0.5 mL of each sample filtrate, standard or blank (distilled water), was mixed with 1.5 mL of 95% ethanol, 0.1 mL of 10% AlCl_3_, 0.1 mL of 1 M potassium acetate, and 2.8 mL of deionized water. After a 30‐min incubation at room temperature, the absorbance was measured at 415 nm using an ultraviolet–visible (UV–Vis) spectrophotometer.

### 2.6 Determination of Beta‐carotene and Lutein content

Beta‐carotene and lutein were quantified, according to the method described by Bhatnagar‐Panwar et al. (2015). A β‐carotene standard stock solution (10.9 mg/100 mL) was prepared in n‐hexane and further dilutions were made with n‐hexane to yield final concentrations ranging from 1.9 to 10.9 mg/mL. Similarly, lutein standards ranging from 3 to 30 μg/mL were used to generate calibration curve. Each vegetable sample (200 mg) was mixed with 8 mL ethanol containing 0.5% (v/w) butylated hydroxytoluene (BHT) and vortexed for 1 min before adding 2 mL of hexane:acetone (2:1) solution. The mixture was incubated for 10 min in the dark at RT and then centrifuged at 5000 rpm for 10 min at 4°C. The supernatant was transferred to a fresh tube and an equal volume of methanol containing 0.5% BHT and 15% KOH was added, incubated at 80°C for 15 min in a water bath, and then immediately chilled on ice for 5–10 min for saponification to occur. A solution of 4 mL distilled water and 3 mL petroleum ether containing 0.5% BHT was added to the saponified extract for better phase separation. The mixture was centrifuged at 5000 rpm for 10 min at 4°C, followed by transfer of the upper colored organic phase to a fresh tube. The leftover residue was re‐extracted twice with 4 mL of petroleum ether containing 0.5% BHT, and the upper phases were pooled and dried in a rotary vacuum evaporator. The residue containing the carotenoid was suspended in 2 mL of mobile phase solvent consisting of methanol:acetonitrile:chloroform (50:40:10) with 0.5% BHT and filtered through a 0.45‐μm syringe filtered into an amber‐colored HPLC vial and immediately analyzed by HPLC by using a ultraviolet (UV) detector. Aliquots of 20 μL were injected into an Agilent reverse‐phase C18 (5 μ, 4.6 mm × 250 mm) column at 30°C. A flow rate of 0.7 mL/min was maintained, and the elution of beta‐carotene was monitored at 450 nm. Peaks were identified by their retention time and quantified against the standard curve.

### 2.7 Determination of glucoraphanin and sulforaphane content

Using the method described by Bello et al. (2018) and Sivakumar et al. (2007), exactly 0.5 g of freeze‐dried ground kale samples was extracted in 12.5 mL of 70% methanol (sample‐to‐solvent ratio of 1:25 w/v) in a water bath at 70°C for 30 min before centrifuging at 4000 rpm for 30 min at 4°C. The supernatant was dried by a rotary vacuum evaporator at 40°C. Dried samples were dissolved in 3 mL HPLC‐grade water and filtered through 0.45‐μm syringe filters. Glucoraphanin and sulforaphane were quantified according to the method described by Celik et al. (2014). The mobile phases were acetonitrile:water, 30:70 v/v for sulforaphane and acetonitrile:water:formic acid, 1:99:0.1 v/v for glucoraphanin. To prepare the standards, 5 mg each of sulforaphane and glucoraphanin (Sigma Aldrich, St. Louis, MO, USA) was dissolved in 10 mL acetonitrile to create a stock solution that was serially diluted in the mobile phase to make six standards ranging from 50 to 500 μg/mL. Exactly 10 μL of the standards and samples was injected into a reverse‐phase C18 (3 μm, 150 mm × 3 mm) column (ESA Biosciences, Inc.) with a flow rate of 0.3 mL/min with detection by an ultraviolet (UV) detector in 202 nm for glucoraphanin and 230 nm for sulforaphane at RT.

### 2.8 Determination of antinutritional factors

#### 2.8.1 Oxalate content

Oxalate present in the samples was extracted with hydrochloric acid (HCl) and then reacted with calcium chloride (CaCl_2_) to convert it to calcium oxalate precipitate, as described by Ranganna (2007). Exactly 2.0 g of sample was mixed with 25 mL of 1.5% HCl solution, heated in a water bath at 95°C for 10 min, cooled, and filtered through Whatman No. 1 filter paper. The filtrate was treated with 10 mL of 0.025M CaCl_2_, mixed/stirred for 5 min, and left at RT for 30 min to allow calcium oxalate to precipitate. Using a pre‐weighed dry filter paper and crucible, the sample was filtered, the filter paper was placed into the crucibles and dried in a hot air oven at 100–105°C to constant weight. Oxalate content was calculated by estimating the difference in the weight of filter paper and expressed as milligrams per gram (mg/g) of sample dry weight.

#### 2.8.2 Tannin content

The tannin content was determined by the Folin–Ciocalteu assay, as described by Ranganna (2007). Exactly 0.5 g of homogenized sample was added to 50 mL of deionized water and boiled for 30 min. Then the mixture was transferred into 50 mL centrifuge tubes and centrifuged at 3000 rpm for 10 min at 4°C and supernatant collected. A tannic acid standard stock solution of 10 mg/mL was prepared by dissolving 100 mg tannic acid in 10 mL of 80% ethanol, which was serially diluted to prepare standards of 0.1, 0.2, 0.3, 0.4, 0.5, and 1.0 mg/mL. In test tubes, 100 μL of the filtered extracts were mixed with 0.5 mL of Folin–Ciocalteu reagent, 1 mL of Na_2_CO_3_ (75 g/L), and 7.5 mL deionized water was added, vortex mixed, and incubated for 40 min in dark at room temperature. Absorbance of the standards and samples were measured at 760 nm using an ultraviolet–visible (UV–Vis) spectrophotometer. From the standard curves of absorbance against the concentration of tannic acid, the amount of tannin present in each sample was calculated as milligrams of tannic acid equivalent per gram of sample dry weight (mg TAE/g dw).

#### 2.8.3 Phytate content

Phytate content was determined according to the method described by Ranganna (2007). Exactly 0.5 g sample was extracted in 10 mL of 2% trichloroacetic acid (TCA) solution with continuous shaking in a plate shaker for 30 min and then centrifuged at 3000 rpm for 5 min. Exactly 1.0 mL of the supernatant was mixed with 1 mL of 0.3% ferric chloride (FeCl_3_) solution and 8 mL of deionized (DI) water and incubated at room temperature for 30 min. Absorbance was determined at 500 nm using an ultraviolet–visible (UV–Vis) spectrophotometer. Amount of phytate was calculated, as shown below:

$$\text{Phytate}\;\left(\mathrm{mg}/g\right)=\left(A\times V\times 1000\right)/\left(E\times W\right)$$

where A is the absorbance of the sample, V is the final volume of the reaction mixture (in mL), E is the extinction coefficient of the phytate–Fe complex [4300 L/(mol·cm) at 500 nm], and W is the weight of the sample in grams.

### 2.9 Determination of antioxidant activity

#### 2.9.1 Extraction of kale extract

Kale samples were extracted using 80% ethanol. Exactly 2.0 g sample was blended with 20 mL of 80% (v/v) ethanol, placed on a shaker for 30 min, vortexed for 1 min, and centrifuged at 4000 rpm and 30°C for 10 min. The supernatant was filtered through Whatman No. 1 filter paper. The process was repeated, and the supernatants were combined and adjusted to a final volume of 50 mL using 80% (v/v) ethanol solvent. The filtrate was stored at −20°C. Antioxidant activity of each extract was determined by two different methods, as shown below.

#### 2.9.2 2,2‐Diphenyl‐1‐picrylhydrazyl (DPPH) free radical scavenging assay

The DPPH assay was performed, according to the method described by Hussain et al. (2016). The ethanolic extract (10 mL) was mixed with a 60 mM solution of DPPH in 2 mL ethanol and kept in the dark at room temperature for 30 min before measuring the absorbance at 517 nm using an ultraviolet–visible (UV–Vis) spectrophotometer. The blank was set to zero using ethanol. The scavenging activity of the extract was expressed as the percentage of inhibition of the DPPH radical, calculated using the formula:

$$\text{Inhibition percentage}\;\left(\mathrm{IP}\right)=\left[\left(A_{\text{control}}-A_{\text{sample}}\right)\times 100\right]/A_{\text{control}}$$

where *A*_control_ is the absorbance of the control (containing all reagents except the sample) and *A*_sample_ is the absorbance of the sample.

### 2.10 Determination of IC_50_ for DPPH activity

To determine the half‐maximal inhibitory concentration (IC_50_) value, 20 mL of the extract obtained above was dried under nitrogen to obtain a solid with precise weight. A 1.0% solution of the dried extract was prepared in methanol. Volumes ranging from 1 to 10 mL were transferred to six different test tubes and 2 mL of DPPH solution was added to each. The control contained only the DPPH solution. The samples were vigorously shaken and then kept in the dark at RT for 30 min. Absorbance was measured at 517 nm using an ultraviolet–visible (UV–Vis) spectrophotometer. The IC_50_ representing the concentration of the extract that caused a 50% reduction in DPPH absorbance was determined by analyzing the absorbance versus concentration graph, as shown by Hussain et al. (2016).

#### 2.10.1 2,2′‐azinobis‐(3‐ethylbenzothiazoline‐6‐sulfonic acid diammonium salt) (ABTS) assay

The radical scavenging capacity of each sample extract was determined by estimating its ability to scavenge ABTS free radicals using the method described by Yu et al. (2018). Free radicals were generated by oxidizing ABTS with manganese dioxide at RT. Briefly, the ABTS stock solution was prepared by dissolving 0.2205 g of ABTS chromophore diammonium salt in 80 mL of deionized water while stirring with a magnetic bar and then the manganese dioxide powder was slowly mixed in the solution with a spatula until the pale green color changed into deep blue–green color. The stock solution was filtered and diluted with phosphate buffer to prepare a working solution with an absorbance of 0.70 ± 0.005 at 734 nm (UV–Vis). Trolox solution at 1.25 to 75 μg/mL was used as the standard curve. A solution of 2 mL phosphate buffer in 160 μL DI water was used as the blank. The reaction mixture comprised 160 μL of standard sample with 2 mL of ABTS. The mixture was vortexed for 30 s and after 90 s reaction time, the absorbance was read at 734 nm using an ultraviolet–visible (UV–Vis) spectrophotometer.

#### 2.10.2 In vitro assessment of anti‐inflammatory properties

For the in vitro anti‐inflammatory assay, only three fermentation groups were evaluated; unfermented kale was compared with traditional fermentation (2% salt w/w), and kale fermented with mixed cultures of *L. lactis* and *L. acidophilus*. Kale samples extracted in 80% methanol, as described above, were dried using a rotary vacuum evaporator (at 40°C). The dried extracts were weighed and dissolved in dimethyl sulfoxide (DMSO) to make a final concentration of 100 μg/mL. The sample groups used for this assay are presented in Table 2.

**TABLE 2:** Treatments used for in vitro anti‐inflammatory assays.

Treatment	Definition	Kale extract definition
Cell media with DMSO	Negative control	—
LPS	Positive control	—
LPS + F0 extract	Test group	Unfermented
LPS + F1 extract	Test group	Natural fermentation
LPS + F5 extract	Test group	LAB fermentation (*L. lactis* & *L. acidophilus*)

#### 2.10.3 Cell culture and treatment

Murine RAW 264.7 macrophages (ATCC, Manassas, VA, USA) were cultivated in high‐glucose Dulbecco's Modified Eagle's Medium (DMEM) supplemented with 10% fetal bovine serum (FBS) (Cytiva HyClone, USA) and 100 units/mL penicillin (Gibco, NY, USA). The dose of kale extract used (100 μg/mL) was based on previous studies by Hwang and Lim (2014) and Jeong et al. (2018). In 6‐well plates, 6.8 × 10^5^ cells were seeded in 1 mL of cell culture medium and incubated at 37°C in a humidified atmosphere containing 5% carbon dioxide (CO_2_). After 2 days, confluent cells were treated with 100 μg/mL of kale extract or DMSO for 4 h followed by washing with cold phosphate‐buffered saline (PBS) and stimulated with (1 μg/mL) pro‐inflammatory lipopolysaccharide (P‐LPS) produced by *E. coli* O111:B4 (Sigma‐Aldrich, St. Louis, MO, USA) for 20 h. Two replicates were used for each of the sample extracts, resulting in a total of six readings for each sample group. The treatments are summarized in Table 2.

#### 2.10.4 RNA isolation and cDNA synthesis

Cells were washed with ice‐cold PBS and scrapped in 1 mL TRIzol. Contents were homogenized in 250 μL chloroform and centrifuged at 10,000 rpm for phase separation. The top layer was collected, mixed with 550 μL isopropanol, and centrifuged at 13,000 rpm for 20 min. The pellet was washed with 1 mL of 75% ethanol and centrifuged at 9500 rpm. The pellet was redissolved with 25 μL nuclease‐free water in 1.5 mL Eppendorf tubes. RNA concentration was determined using the Qubit RNA BR Assay kit on the Qubit 4 fluorometer (Thermo Fisher Scientific, MD, USA). About 2000 ng of RNA from each sample was used for complementary DNA (cDNA) synthesis using High‐Capacity cDNA Reverse Transcription kit (Thermo Fisher Scientific, MD, USA). The cDNA was stored at −80°C for further qPCR applications.

#### 2.10.5 Analysis of target genes by qPCR

Genes encoding pro‐inflammatory cytokines such as interleukin‐1 beta (IL‐1β), interleukin‐6 (IL‐6), tumor necrosis factor‐alpha (TNF‐α), and inducible nitric oxide synthase (iNOs) were determined by quantitative polymerase chain reaction (qPCR). Primer sets for these genes and β‐actin (all from Integrated DNA Technologies IDT, Coralville, IA, USA) used are shown in Table 3. Samples contained 10 ng cDNA and 10 μM primers in Applied Biosystems 2X PowerUp SYBR Green Master Mix (Thermo Fisher Scientific, MD, USA) in a final volume of 10 μL. Reverse transcription polymerase chain reaction (RT‐PCR) cycling conditions on the CFX 96 (Bio‐Rad Laboratories Inc., CA, USA) were 2 min at 50°C, 2 min at 95°C, followed by 40 cycles of two‐step denaturation at 95°C for 15 s and annealing extension at 60°C for 1 min. The relative amount of target mRNA in each sample was normalized to β‐actin, the endogenous control gene. Data were analyzed according to the 2^−ΔΔCT^ method, and fold difference was calculated regarding LPS control.

**TABLE 3:** Primer sequences used for qPCR.

Gene	Forward	Reverse
iNOS	CAC CTT GGA GTT CAC CCA GT	ACC ACT CGT ACT TGG GAT GC
TNF‐a	TAC TGA ACT TCG GGG TGA TTG GTC C	CAG CCT TGT CCC TTG AAG AGA ACC
IL‐1ß	CCA GCT TCA AAT CTC ACA GCA G	CCA GCT TCA AAT CTC ACA GCA G
IL‐6	TCC AGT TGC CTT CTT GGG AC	GTA CTC CAG AAG ACC AGA GG
β‐actin	CCA GAG CAA GAG AGG TAT CC	CTG TGG TGG TGA AGC TGT AG

### 2.11 Determination of microbiota composition in vegetable samples

To evaluate the effects of fermentation on the surface microbiota composition, we extracted DNA from the vegetable samples and used 16S sequencing to determine bacterial composition.

#### 2.11.1 DNA extraction and amplification of the 16S hypervariable regions

To extract DNA, 100 mg of each freeze‐dried powdered vegetable sample was homogenized in lysing buffer using the FastPrep‐24 (MP Biomedicals, OH, USA) and DNA was extracted according to manufacturers' instructions given in the DNeasy PowerLyzer PowerSoil kit (Qiagen, MD, USA). DNA concentration was determined on the Qubit 4.0 Fluorometer using the Qubit™ dsDNA BR assay kit (Thermo Fisher Scientific, MD, USA). To target bacterial DNA, seven hypervariable regions of the 16S gene, i.e., V2, V3, V4, V6, V7, V8, and V9 were amplified by polymerase chain reaction (PCR) using reagents and the manufacturer's protocol provided in the Ion 16S™ Metagenomics Kit (Thermo Fisher Scientific, MD, USA). Using Agencourt AMPure XP beads (Beckman Coulter, Nyon, Switzerland) on the DynaMag™‐2 magnetic rack, the amplification products were purified to remove primer dimers and small mispriming products, washed with fresh 70% ethanol, and then eluted into 15 μL of nuclease‐free water. The purified PCR product was quantified by the Qubit™ dsDNA BR Assay Kit before calculating DNA for library preparation.

#### 2.11.2 Library preparation, template preparation, and sequencing

Libraries were prepared and barcoded using end repair buffer enzymes contained in the Ion Plus Fragment Library Kit and the Ion Xpress™ Barcode Adapters 33‐53 (Thermo Fisher Scientific, Rockville, MD, USA). Samples were purified using Agencourt AMPure XP beads, washed with fresh 70% ethanol, and then eluted into 20 μL of low TE (Tris–EDTA) in 1.5 mL Eppendorf tubes. The library concentration was determined by quantitative polymerase chain reaction (qPCR) using the Ion Universal Library Quantitation Kit (Thermo Fisher Scientific, MD, USA). Libraries were diluted to yield 10 pM inputs for template preparation and enrichment using an Ion OneTouch 2 System and the Ion PGM Hi‐Q OT2 Kit. After template enrichment with the Ion OneTouch2, template‐positive Ion PGM Hi‐Q Ion Sphere Particles (ISPs) with 400 base‐pair average insert libraries were used for sequencing on a 530 chip using the Ion GeneStudio S5 system (Thermo Fisher Scientific, Grand Island, NY, USA).

#### 2.11.3 Microbiome data diversity analysis

Fastq sequence files were curated from the Ion Reporter and processed using Microseq as we have done before (Shahinozzaman et al., 2021). Amplicon sequence variants (ASVs) were determined. Forward and reverse reads were evaluated as single reads and then merged to produce full amplicons. Data were rarefied to a sampling depth of 10,000 reads per sample and a phylogenetic tree was built with FastTree. Taxonomic classification was performed using the SILVA v138 database and the MicroSEQ database v2013.1.

Taxonomic classification was performed based on relative abundance and compared between each sample group. Using the Galaxy workflow (https://huttenhower.sph.harvard.edu/galaxy), we determined the taxa most likely to explain differences between the seven groups using linear discriminant analysis (LDA) effect size (LEfSe) that utilizes a non‐parametric Kruskal–Wallis rank‐sum test to assess differential features with significantly different abundances between assigned taxa and performs LDA to estimate the effect size of each sequence variant. LDA scores ranking differential taxa are displayed on a bar chart, according to their effect size. A significant alpha level of .05 and an effect size threshold of four times greater difference were used for displaying results (Figure 9E).

### 2.12 Statistical analysis

All experiments were carried out in triplicate. Data are expressed as mean ± standard error of the mean. Statistical analysis was performed by one‐way analysis of variance (ANOVA). Any significant differences were further tested by a post hoc test (Tukey test) using GraphPad Prism 9 (San Diego, CA, USA). *p* values <.05 were considered statistically significant.

## 3 RESULTS

### 3.1 Physicochemical analysis (pH and % titratable acidity)

**FIGURE 2:**
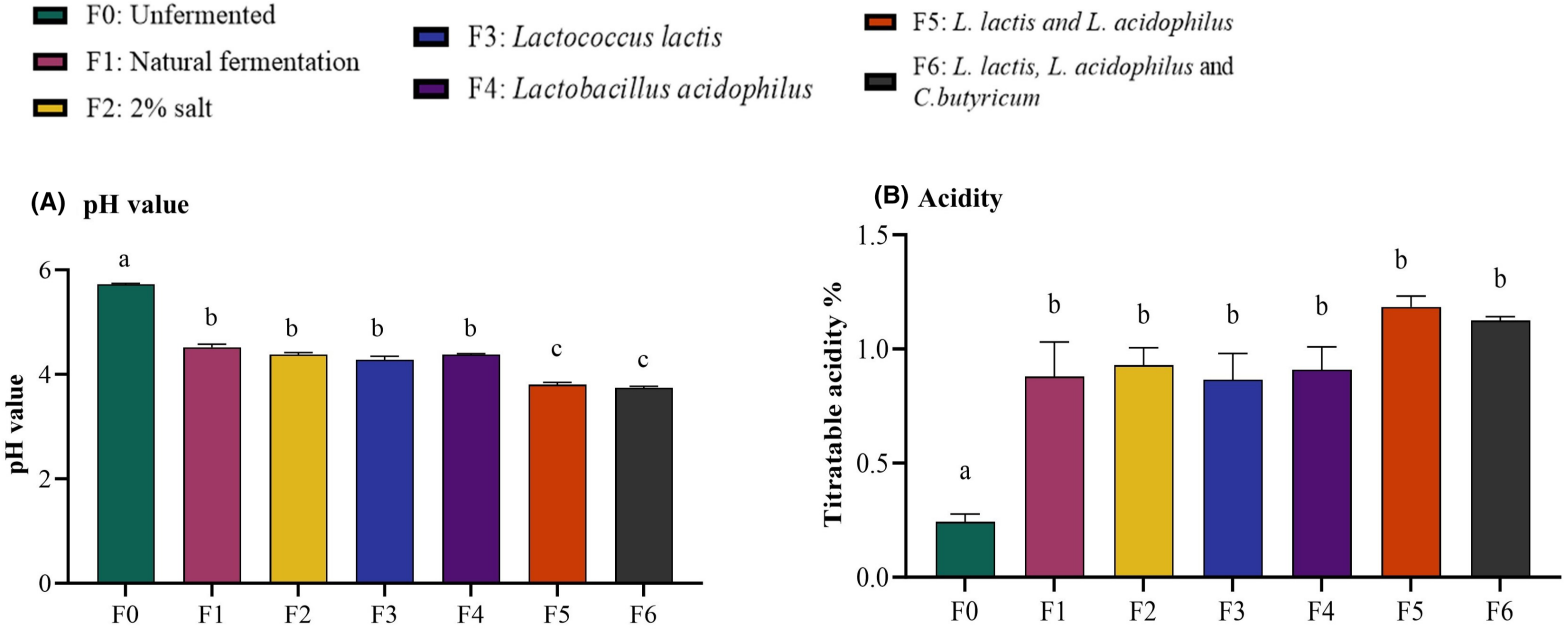
Fermentation lowered pH and increased titratable acidity. pH and titratable acidity were determined before and after 16 days of fermentation of each group. (A) pH significantly decreased in fermented kale. (B) Titratable acidity (as % lactic acid) significantly increased in fermented kale. Results are shown as mean ± SEM, ‘a’ – Different letters indicate significant difference between treatments (*p* < .05; *n* = 3).

The pH and % titratable acidity were determined before fermentation and post‐fermentation. The average pH of unfermented kale was 5.72 ± 0.04 and it decreased after fermentation in all groups (Figure 2A; *p* < .05, *n* = 3). Among the fermentation groups, pH values ranged from 4.52 (natural fermentation) to 3.74 (*L. lactis*, *L. acidophilus*, and *C. butyricum*) (Figure 2A). Titratable acidity significantly increased following fermentation in all groups compared to the unfermented group. The mixed cultures of *L. lactis* and *L. acidophilus* induced the highest increase in titratable acidity, but the increase was not statistically different from those of all other fermentation groups (Figure 2B).

### 3.2 Total polyphenol content

**FIGURE 3:**
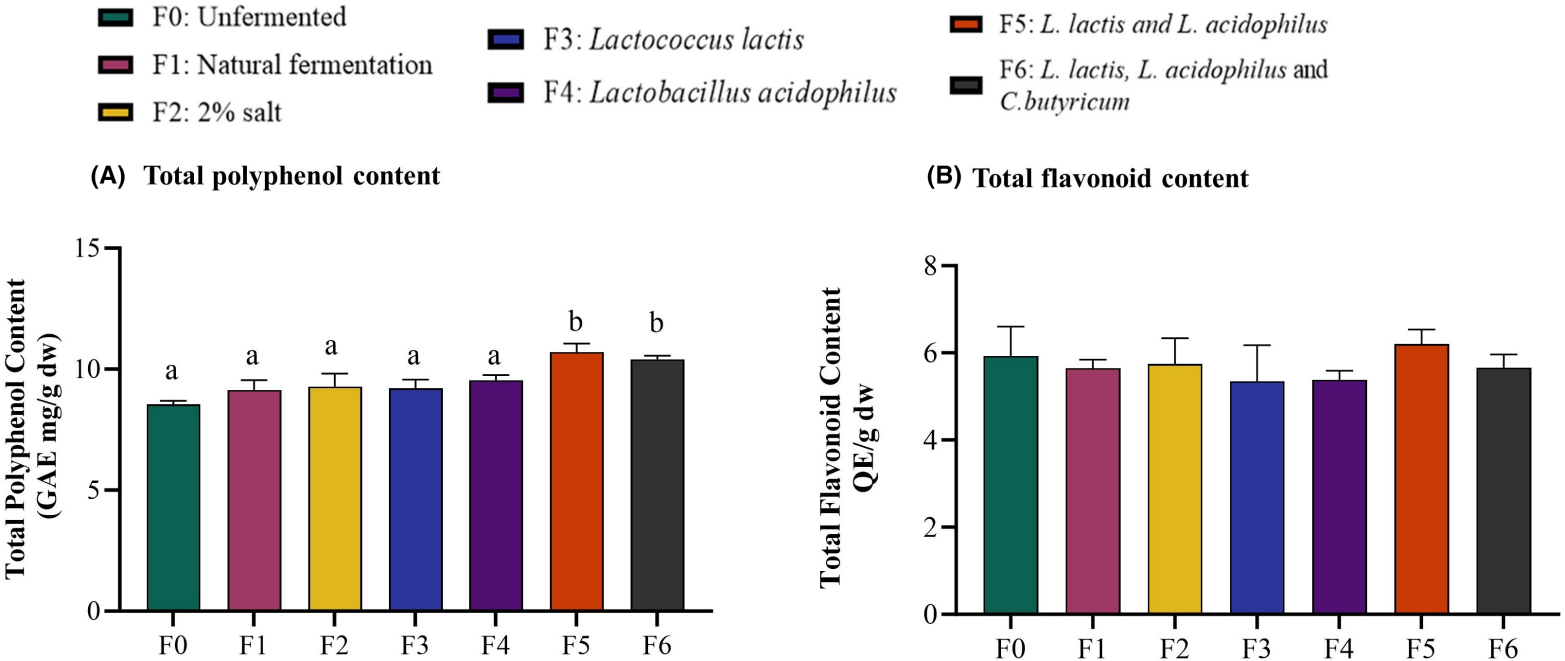
Fermentation using mixed cultures of lactic acid bacteria increased the total polyphenol content but not total flavonoids. Total polyphenol and flavonoid contents were quantified using the Folin–Ciocalteu spectrophotometric method and aluminum trichloride colorimetric method, respectively. (A) Total polyphenol content was significantly higher in kale fermented with mixed culture of *L. lactis* and *L. acidophilus*. (B) Total flavonoid content expressed as quercetin equivalent was not different between fermented and unfermented kale (*p* = ns). Results are shown as mean ± SEM. ‘a’ – Different letters indicate significant difference between treatments (*p* < .05; *n* = 3).

Total polyphenol content ranged between 8.5 and 10.7 mg GAE/g (Figure 3A). Unfermented samples had the lowest concentration, while the highest concentration was observed in the fermentation group with mixed cultures of *L. lactis* and *L. acidophilus*. All fermentation types showed a slight increase in total polyphenols compared to unfermented kale, but statistically significant increases were observed only in the groups with mixed bacterial cultures (F5 and F6 with mixed LAB and *C. butyricum*).

### 3.3 Total flavonoid content

The total flavonoid content determined as quercetin equivalent (QE) varied from 5.35 to 6.20 mg QE/g dry sample (Figure 3B). No significant differences were observed between the unfermented and fermented kale (*p* = ns). However, the mixed cultures of *L. lactis* and *L. acidophilus* recorded the highest flavonoid content 6.20 mg QE/g.

### 3.4 Glucosinolates (glucoraphanin and sulforaphane content)

**FIGURE 4:**
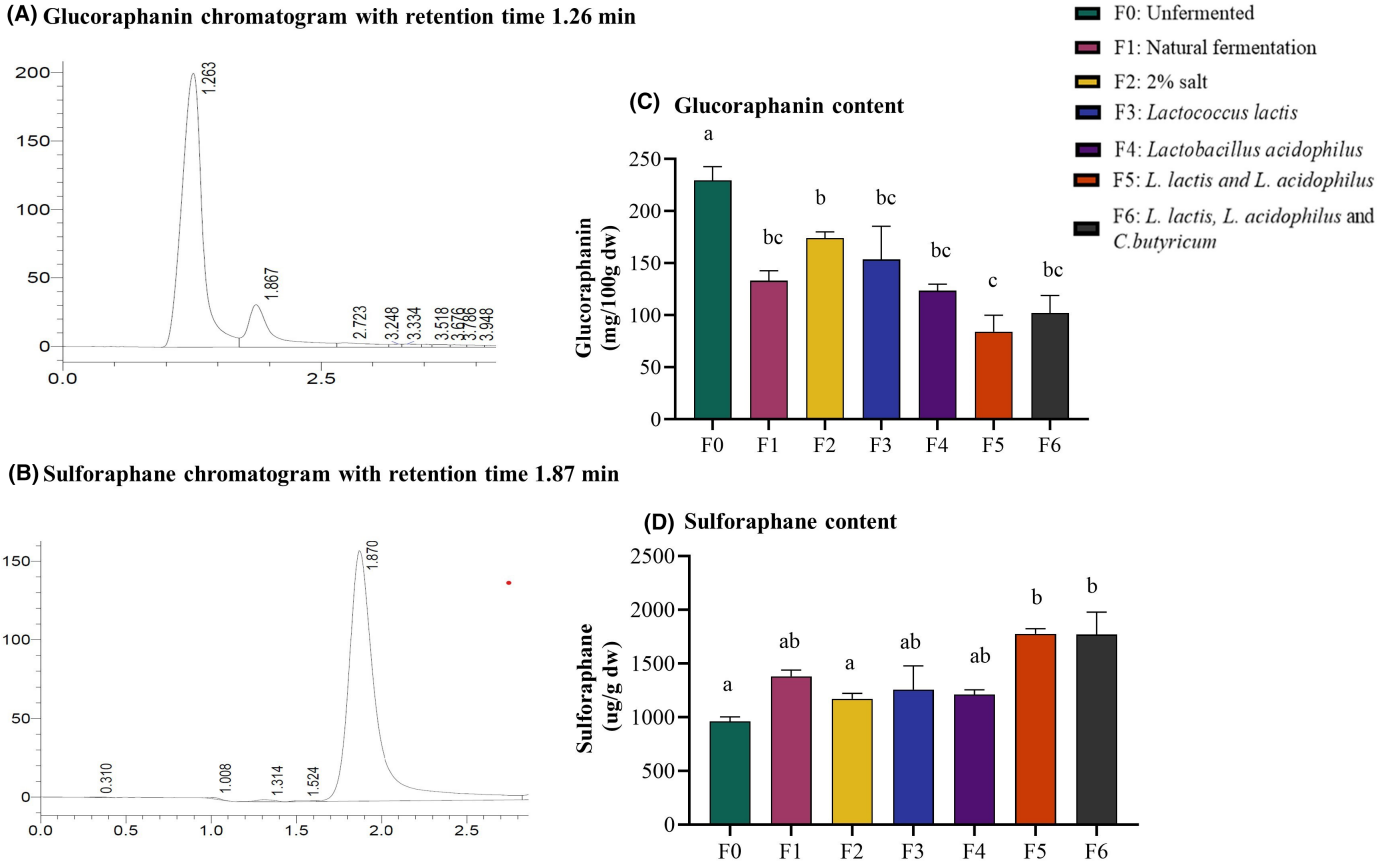
Fermentation decreased glucoraphanin content but increased sulforaphane content in kale. Methanolic extracts of kale were analyzed for glucoraphanin and sulforaphane content by HPLC coupled to an ultraviolet (UV) detector. (A) and (B). HPLC chromatograms of glucoraphanin and sulforaphane (C). Glucoraphanin significantly decreased in all fermented groups, but fermentation with *L. lactis* and *L. acidophilus* was the most effective in reducing it. (D). Sulforaphane increased in all fermented groups, but fermentations with *L. lactis* and *L. acidophilus* were the most effective in increasing it. Results are shown as mean ± SEM. ‘a’ – Different letters indicate significant difference between treatments (*p* < .05; *n* = 3).

The glucoraphanin contents ranged between 84.02 and 229.4 mg/100 g. The sulforaphane contents ranged from 960.8 to 1777 μg/g, respectively. Glucoraphanin was highest in the unfermented kale and significantly decreased in all fermented groups (*p* < .005; *n* = 3) (Figure 4A,C). Sulforaphane significantly increased in all fermented groups compared to unfermented kale, except in the group fermented with 2% salt (Figure 4B,D). Group F5 with mixed cultures of *L. lactis* and *L. acidophilus* had the lowest glucoraphanin at 84.02 μg/g and the highest sulforaphane content at 1777 μg/g; it was the most effective in the conversion of glucoraphanin to sulforaphane. Although all fermentations resulted in the conversion of glucoraphanin to sulforaphane, the 2% salt fermentation was the least effective (Figure 4A,B). While fermentation by *L. lactis* and *L. acidophilus* reduced glucoraphanin by about 50%, the sulforaphane was increased by about 50% compared to the unfermented control in the same samples.

### 3.5 Beta‐carotene and Lutein content

**FIGURE 5:**
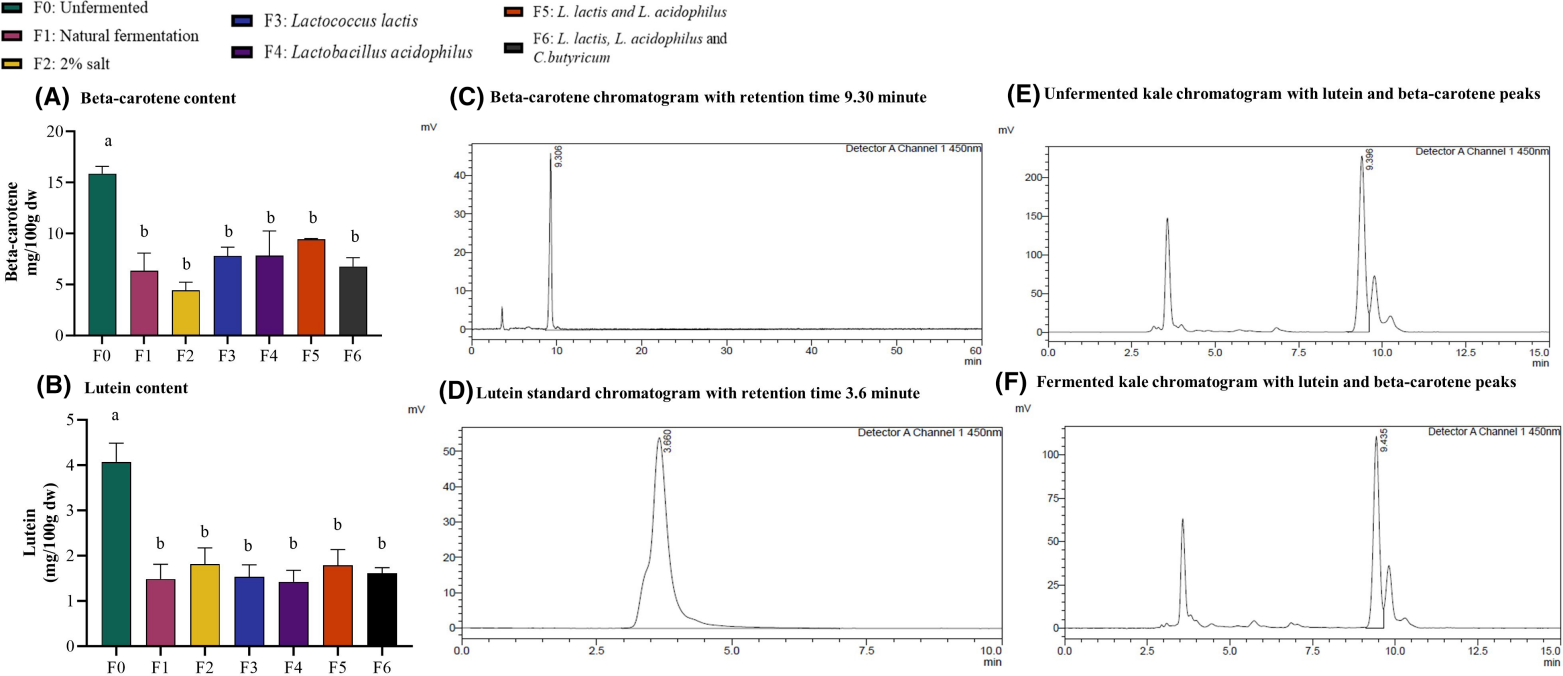
Fermentation decreased beta‐carotene and lutein content in kale. Carotenoid extracts of kale were analyzed for beta‐carotene and lutein content by HPLC coupled to an ultraviolet (UV) detector at 450 nm. (A, B). Beta‐carotene and lutein content. (C, D). HPLC chromatogram of beta‐carotene and lutein standards. (E). HPLC chromatogram of carotenoid extracts from unfermented kale. (F). HPLC chromatogram of carotenoid extracts from fermented kale (F1). Results are shown as mean ± SEM, ‘a’ – Different letters indicate significant difference between treatments (*p* < .05; *n* = 3).

Beta‐carotene levels ranged from 4.4 to 15.8 mg/100 g. All fermentation methods significantly lowered beta‐carotene levels. Unfermented kale had the highest beta‐carotene content at 15.9 mg/100 g followed by fermentation with mixed cultures of *L. lactis* and *L. acidophilus* at 9.42 mg/100 g. The group fermented with 2% salt had the lowest beta‐carotene at 4.4 mg/100 g. However, the decrease among all fermented groups was not significantly different (Figure 5A). Lutein content ranged between 1.42 and 4.07 mg/100 g. Lutein content also significantly decreased in fermented kale, irrespective of the fermentation method (Figure 5B).

### 3.6 Effect of fermentation on antinutritional factors

#### 3.6.1 Oxalate content

**FIGURE 6:**
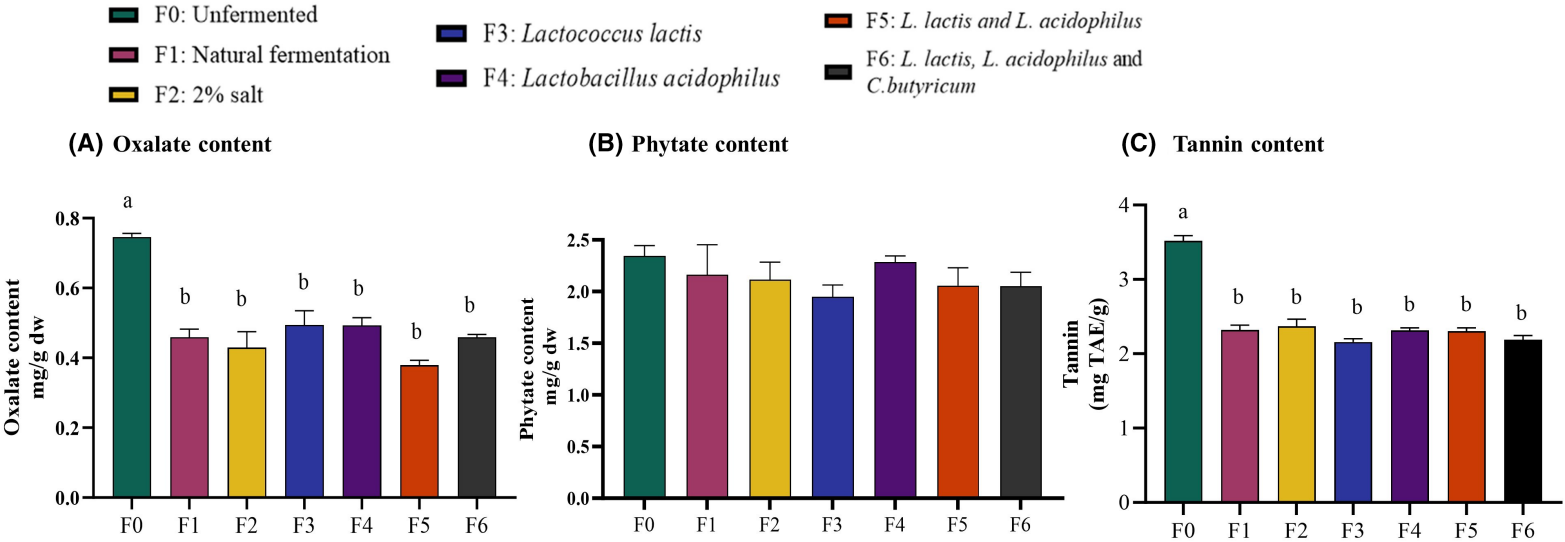
Fermentation decreased oxalate and tannin but not phytate content. Oxalate was extracted with HCl and then reacted with CaCl_2_ to convert it into calcium oxalate precipitate. Tannin content was determined by Folin–Ciocalteu spectrophotometric assay using tannic acid standard. Phytate was quantified using a spectrophotometric assay. (A) Oxalate content significantly decreased in all fermentation groups (*p* < .05; *n* = 3). (B) Phytate content was not different (*p* = ns) between fermented and unfermented kale. (C) Tannin content significantly decreased in all fermentation groups compared to unfermented kale (*p* < .0001; *n* = 3). Results are shown as mean ± SEM. ‘a’ – Different letters indicate significant difference between treatments (*p* < .05; *n* = 3).

The amount of oxalate in unfermented kale was 0.75 ± 0.01 mg/g. All fermentation methods decreased oxalate content, compared to the unfermented group (*p* < .05; *n* = 3). Group (F5) with mixed cultures of *L. lactis* and *L. acidophilus* reduced the oxalate content the most by 49%, as shown in Figure 6A. However, the % reduction was not different among all fermentation methods.

#### 3.6.2 Phytate content

The amount of phytate in unfermented kale was 2 mg/g dw. All fermentation methods slightly decreased the phytate content, but statistical significance was not reached. Fermentation with *Lactococcus lactis* yielded the lowest phytate content at 1.95 mg/g dw (Figure 6B).

#### 3.6.3 Tannin content

Unfermented kale contained 9.05 mg/g of tannin. Although all fermentation processes decreased tannin content compared to unfermented kale, significant decreases were observed only in natural fermentation and F5 with mixed cultures of *L. lactis* and *L. acidophilus* at 46% and 22% reduction, respectively (Figure 6C).

### 3.7 Effect of fermentation on the antioxidant capacity of kale

#### 3.7.1 DPPH free radical scavenging assay

**FIGURE 7:**
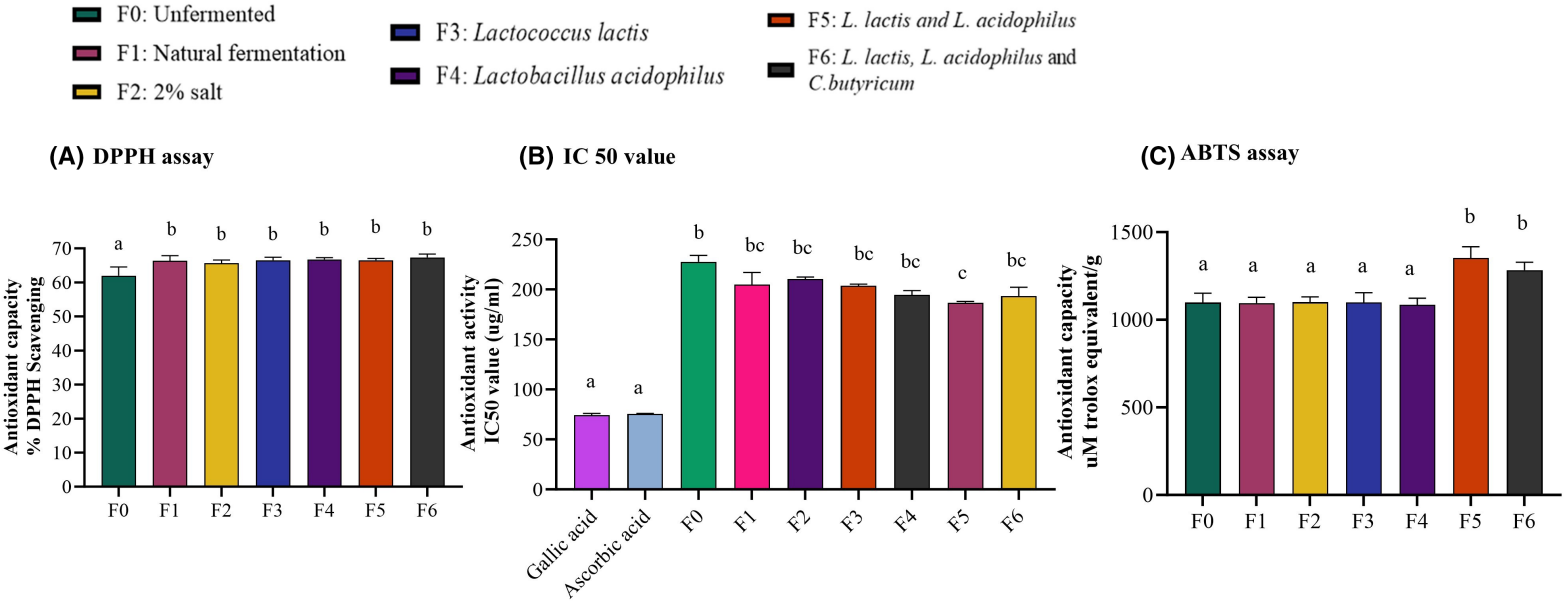
Fermentation increased the antioxidant capacity of kale. Ethanolic extracts of kale were evaluated for antioxidant capacity by the DPPH and ABTS free radical scavenging assays. (A). All fermentation types increased antioxidant capacity, as determined by the DPPH assay. (B). Compared to the control, only F5 (*L. lactis* and *L. acidophilus)* fermentation reduced the IC_50_ value. (C). Compared to the unfermented control, only F5 and F6 (with *L. lactis* and *L. acidophilus)* reduced the IC_50_ value. Compared to the unfermented control, only F5 and F6 (groups with *L. lactis* and *L. acidophilus*) enhanced antioxidant capacity, as determined by ABTS radical scavenging assay. Results are shown as mean ± SEM. ‘a’ – Different letters indicate significant difference between treatments (*p* < .05; *n* = 3).

Unfermented kale demonstrated the lowest antioxidant activity at 61.99%. All fermentation methods significantly increased antioxidant activity (*p* < .05; *n* = 3). The increase was not different between all fermentation methods (Figure 7A). The IC_50_ values of kale extracts ranged between 186.7 and 220.9 μg/mL, while that of ascorbic acid and gallic acid was 75.56 ± 0.29 μg/mL, almost 3 times lower than that of unfermented kale. Only fermentation of the mixed culture of *L. lactis* and *L. acidophilus* reduced IC_50_ values compared to unfermented kale (Figure 7B; *p* < .05). All other fermentation groups did not have a lower IC50 compared to unfermented kale.

#### 3.7.2 ABTS free radical scavenging capacity

The antioxidant capacity evaluated against ABTS free radicals ranged between 1096 and 1354 μM TE/g. Only fermentation groups F5 and F6 (mixed culture of LAB and LAB*+ C. butyricum*) increased the ABTS scavenging activity compared to the unfermented control (Figure 7C, *p* < .05; *n* = 30). The other fermentation groups did not change ABTS scavenging ability compared to the control (*p* = ns).

### 3.8 Effect of fermentation on anti‐inflammatory properties of kale

Cells were treated with kale extract for 4 h before stimulating them to induce inflammation using LPS for 20 h. The inhibition of iNOs, IL‐1β, IL‐6, and TNF‐α by kale extracts (F0, F1, and F5) was expressed as a percentage of the control group (LPS only). The expression of cytokines in the LPS control was set at 100%. The results showed a significant reduction in the gene expression between all four cytokines by all three kale extracts tested (*p* < .005; *n* = 4). There was no statistically significant difference between fermented and unfermented kale performance in reducing inflammation.

**FIGURE 8:**
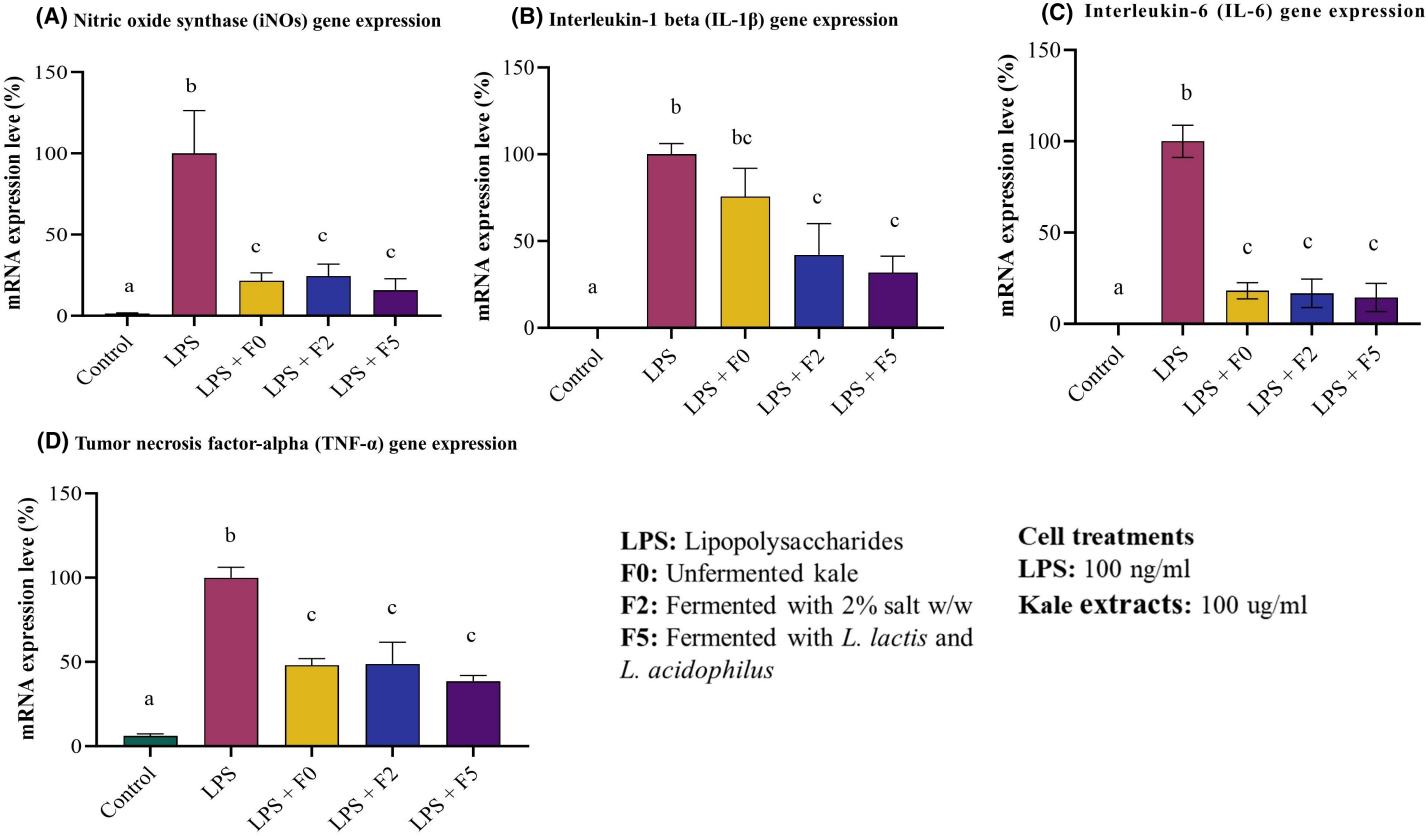
All kale extracts protected cells from LPS‐induced inflammation. The ability of ethanolic extracts to protect against inflammation was compared by determination of inflammatory cytokine expression induced by LPS in RAW 264.7 macrophages. (A) All kale extracts significantly reduced iNOs expression. (B) The extracts from fermented kale reduced (IL‐1β) expression compared to the positive control. However, the unfermented kale extract did not reduce expression compared to LPS control. (C) All kale extracts significantly reduced IL‐6 expression compared to LPS positive control. (D) All kale extracts significantly reduced TNF‐α expression compared to the positive control. Results are shown as mean ± SEM. ‘a’ – Different letters indicate significant difference between treatments (*p* < .05; *n* = 6).

Expression of iNOS was significantly reduced to 15.7% by LAB‐fermented kale, followed by unfermented kale (21.6%) and traditionally fermented kale (24.4%) (Figure 8A). Expression of IL‐1β was reduced to 31.9% by LAB‐fermented kale, followed by traditionally fermented kale (42.0%) and unfermented kale (75.7%) (Figure 8B). IL‐6 was reduced to 14.5% by LAB‐fermented kale, followed by traditionally fermented kale (16.7%) and unfermented kale (18.2%) (Figure 8C). TNF‐α expression was significantly reduced to 38.4% by LAB‐fermented kale, followed by unfermented kale (48.1%) and traditionally fermented kale (48.8) (Figure 8D).

#### 3.8.1 Comparative analysis of the surface microbiota of kale

The composition of the surface microbiota was determined by 16S sequencing.

##### Phylum Proteobacteria

**FIGURE 9:**
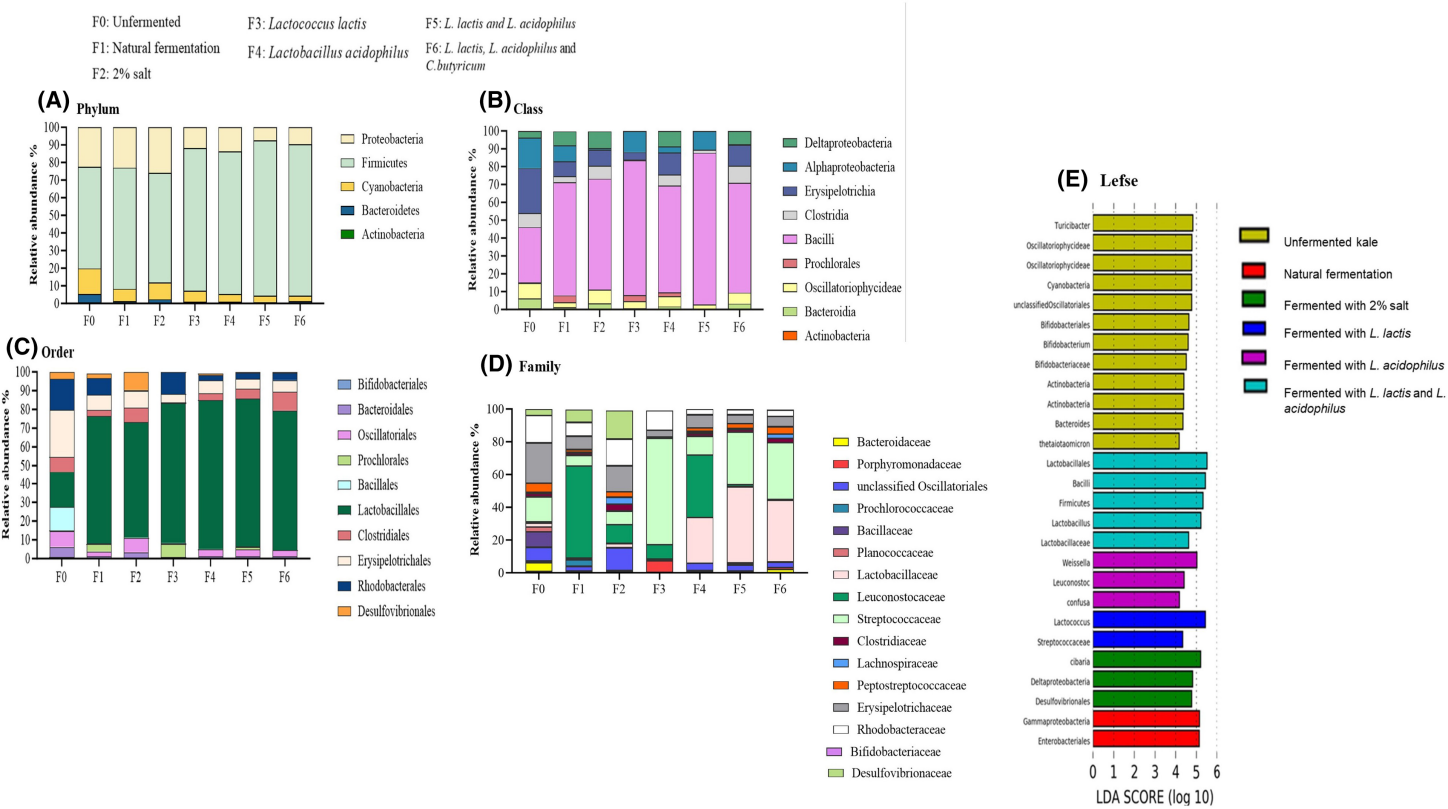
Surface microbiota composition of kale. Bacterial DNA extracted from each sample was used to sequence the 16S gene. Fastq sequence files were used for bioinformatics analysis in QIIME2 to determine diversity and taxa abundance. Analysis by LeFSE (linear discriminant analysis effect size) was used to determine the taxa that contribute most to the differences between groups. (A–D) Abundance of bacterial taxa at phylum, class, order, and family levels. (E) Linear discriminant analysis (LDA) scores.

The most significant change induced by fermentation was a decrease in the phylum Proteobacteria. Proteobacteria were present at 24.8% in the unfermented group. Fermentation by natural methods (F1 and F2) had no significant impact on the proportions of Proteobacteria. All fermentation groups involving lactic acid bacteria (F3, F4, F5, and F6) significantly decreased the representation of Proteobacteria (Figure 9A). At class levels, Proteobacteria consisted mainly of Alphaproteobacteria and Deltaproteobacteria (Figure 9B). While the orders under this phylum were Desulfovibrionales and Rhodobacterales (Figure 9C). These taxa reduced significantly in fermentation groups involving lactic acid bacteria; in fact, Desulfovibrionales was undetectable in all fermentation groups involving lactic acid bacteria, while Rhodobacterales formed a major proportion of the Proteobacteria in the unfermented group.

##### Phylum Firmicutes

The unfermented kale had 57.6% Firmicutes. After fermentation, Firmicutes increased to between 80.94% and 88.16% in groups inoculated with lactic acid bacteria cultures (F3–F6) compared to unfermented kale. No significant changes in the proportions of this phylum were observed in the F1 and F2, natural fermented groups (Figure 9A). Under Firmicutes, class Clostridia was present at <5%, class Bacilli constituted 31.4%, while class Erysipelotrichia constituted about 25% in the unfermented group. Bacilli increased significantly in all fermented groups, with the highest abundance observed in group F5 (mixed culture of *L. lactis* and *L. acidophilus*) at 85.06%. The bulk of class Bacilli constituted of the order Lactobacillales that increased significantly (almost by 4‐fold) in all fermentation groups compared to the unfermented groups. Under Lactobacillales, Family Lactobacillaceae was only detectable in F4, F5, and F6; the groups fermented with a mixture containing *Lactobacillus acidophilus*. The group fermented with *Lactococcus lactis* had Family Streptococcaceae instead, while Family Leuconostocaceae increased mainly in the natural fermentation group F1 (Figure 9D). Order Bacillales was detectable only in the unfermented groups and reduced in all fermentation groups to undetectable levels. Class Clostridia made up of the order Clostridiales only increased slightly to about 7% in F5 (mixed culture of *L. lactis* and *L. acidophilus and C. butyricum*) (Figure 9C). The class Erysipelotrichia constituted mainly the order Erysipelotrichiales at 25% in the unfermented group. All fermentation types reduced Erysipelotrichia to below 5%.

##### Phylum Bacteroidetes

The unfermented kale had 4.7% Bacteroidetes and all fermentations decreased this representation. In F1 (natural fermentation) and all fermentation groups involving lactic acid bacteria (F3, F4, F5, and F6), Bacteroidetes and associated Bacteroidales were undetectable. Only the F2 with 2% salt fermentation had detectable numbers of Bacteroidales at less than 2%. Family Bacteroidaceae was only detectable in the unfermented group.

##### Phylum Cyanobacteria

The unfermented kale had 14.6% Cyanobacteria, which constituted mainly class Cyanophyceae and order Oscillatoriales. The Cyanobacteria taxa decreased in all fermentation groups (Figure 9A–C).

## 4 DISCUSSION

Compared with all cruciferous vegetables grown and consumed in the United States, kale has been the least studied. Although its popularity is lower compared to broccoli, spinach, or cabbage, the nutraceutical industry has acknowledged kales' superiority; having a denser concentration of several minerals, polyphenols, carotenoids, glucosinolates, and sulforaphane. While the role of kale as a functional food is now well established, our objectives in this study were to investigate how fermentation can further enhance the antioxidant and anti‐inflammatory properties of this food by (i) making the bioactive compounds more accessible, (ii) reducing antinutritional components like oxalate which may cause health issues (iii), and (iv) promoting the prebiotic properties of kale and its function as a delivery vehicle of beneficial bacteria through diet. Furthermore, we sought to compare different well‐established fermentation methods and/or a mix of starter cultures in order to establish the one that confers the most beneficial effects on kale as a functional food.

Our results show that all fermentation methods lowered pH and increased the % titratable acidity. However, only the groups fermented with a mixture of lactic acid producing bacteria (*L. acidophilus and L. lactis*) had increased amounts of total polyphenols and sulforaphane, while fermentation had no effect on flavonoid content. All fermentation methods reduced the amounts of oxalate and tannin contents in kale and enhanced the antioxidant properties of the extract. However, the groups fermented with the mixture of lactic acid producing bacteria acquired even higher antioxidant properties than those fermented with single individual cultures or traditionally without a bacterial inoculum. While kale confers well‐established anti‐inflammatory properties to cells and tissues, fermentation had no impact on this; the unfermented and fermented vegetables conferred equal effects in cell culture. Finally, fermentation conferred important changes in the surface bacterial composition of the vegetable. Specifically, the representation of bacterial taxa from Proteobacteria, a phylum associated with inflammation, was reduced in all groups fermented with lactic acid producing bacteria, while the representation of beneficial Lactobacillales increased. Therefore, the prebiotic properties of the vegetable were enhanced. Fermented kale thus is a symbiotic; it is not only prebiotic but also acts as a vehicle to deliver probiotic bacteria.

Fermentation groups F5 and F6, which involved the combination of *L. lactis* and *L. acidophilus*, resulted in the largest decrease in pH and an increase in titratable acidity (Figure 2) and a significant increase in polyphenols (Figure 3). During fermentation, microorganisms convert the natural sugars in food into lactic acid, lowering its pH and creating an acidic environment that inhibits the growth of harmful bacteria, yeast, and molds while promoting the proliferation of lactic acid bacteria. This contributes to the tangy taste of fermented foods (Vatansever et al., 2017). When vegetables undergo fermentation, their cell wall breakdown results in the release of various compounds, including polyphenols, which may be trapped within the cell structures. Enzymatic activities of microorganisms help release these polyphenols, making them more accessible. Lactic acid bacteria promote esterase enzymatic activity, catalyzing the hydrolysis of ester groups, adding water to break them up to an acid and alcohol. This reaction improves the availability of phenolic acids. Some polyphenols in cruciferous vegetables exist as precursor compounds, and during fermentation, microbial enzymes like these esterases convert these compounds into active polyphenols, increasing the total polyphenol content (Iga‐Buitrón et al., 2023). The esterase activity of lactic acid bacteria improves the availability of phenolic acids by hydrolyzing ester groups, thereby potentially enhancing the health‐promoting properties of these bioactive compounds (De Montijo‐Prieto et al., 2023; Esteban‐Torres et al., 2015; Xiang et al., 2019).

The changes in polyphenol content differ depending on the variety of vegetables, fermentation conditions, microbial strains used, and fermentation duration (Olsen et al., 2010, 2012). Vegetables owe their overall antioxidant activity of polyphenols and higher concentration, or accessibility of polyphenols can help reduce the risk of chronic diseases, such as heart disease, cancer, and diabetes (Zhou & Yu, 2006). The increase of DPPH and ABTS free radical scavenging properties in fermented kale correlated with higher polyphenols and sulforaphane contents in fermented kale (Piao et al., 2005; Rahman et al., 2022). Several studies have found a strong correlation between total phenolic content, ascorbic acid content, flavonoids, and antioxidant capacity (by the DPPH assay). Sulforaphane contributes to free radical scavenging and hence has antioxidant potential (de Figueiredo et al., 2015). Flavonoids, particularly quercetins, are also antioxidants protective against free oxygen radicals (Biswas et al., 2022). While fermentation by all methods had no effect on flavonoid content, the increased polyphenols by lactic acid producing bacteria correlated with an increase in radical scavenging activity of ABTS compared to the other fermentation methods. Lactic acid fermentation can promote the degradation of plant cell walls, facilitating the release or synthesis of diverse antioxidant compounds, production of organic acids, activation of endogenous enzymes, and release of bioactive compounds (de Figueiredo et al., 2015). Flavonoids are a class of secondary plant phenolics with significant antioxidant and chelating properties and are antioxidants protective against free oxygen radicals (Heim et al., 2002). DPPH free radical scavenging assay can also be influenced by amounts of ascorbic acid and other organic acids present in the fermented kale (Scalzo, 2008). Increase in acidity during fermentation can positively influence the antioxidant capacity of fermented foods by microbial hydrolysis, stabilizing antioxidants, enhancing the extraction of bioactive compounds, and increasing the solubility and bioavailability of antioxidants in the final product (Zhao et al., 2021).

All fermentation methods decreased beta‐carotene and lutein up to 72% and 65%, respectively (Figure 5A,B). A similar decrease during fermentation of kale juice was previously reported by Szutowska et al. (2021). During lactic acid fermentation, volatile carotenoid cleavage derivatives are created, contributing to fermented flavor and aroma (Mapelli‐Brahm et al., 2020). Microorganisms can transform carotenoids during fermentation causing variations in the specific carotenoid composition (Szutowska et al., 2020). However, no comprehensive metabolomics studies have reported or profiled carotenoids and their derivatives before and after lactic acid fermentation.

The glucoraphanin content decreased by up to 50% while sulforaphane significantly increased by up to 50% in fermented kale (Figure 4C,D). Fermentation groups with mixed LAB culture had the highest amounts of sulforaphane. Glucoraphanin is converted into sulforaphane by the action of the myrosinase enzyme released during chopping and chewing of cruciferous vegetables (Cai et al., 2019). Sulforaphane is a natural inducer of phase II enzymes in both human and animal bodies, aiding in detoxifying cancer‐causing chemicals. Several factors directly influence sulforaphane formation, including vegetable species, genotype, preharvest conditions, and post‐harvest processing (Gu et al., 2012). Bacteria display myrosinase‐like activity during fermentation, facilitating glucosinolate hydrolysis. The myrosinase‐like activity results in different glucosinolate (GLS) breakdown products (Szutowska et al., 2020). Thus, fermentation made sulforaphane more easily available before consumption and breakdown of kale vegetables in the gut. This eliminates the problem of myrosinase being destroyed during cooking of kale, thus hampering the bioavailability of sulforaphane. Steaming and microwaving have been shown to cause over 90% loss of myrosinase activity, while stir‐frying results in up to 70% loss (Oloyede et al., 2021). This can result in loss of accessibility of sulforaphane, especially in the upper gut. Fermenting kale before cooking eliminates this problem, as sulforaphane is made available before cooking.

Antinutritional factors, such as oxalic, phytic, and tannic acids and their salts, are found in many leafy plant‐based foods including kale and may hinder bioavailability and absorption of essential minerals. Oxalate hinders calcium absorption and has been associated with the formation of kidney stones (Wadamori et al., 2014). Phytate can form insoluble strong chelates with calcium, iron, and zinc, leading to their deficiency. Tannins can bind to dietary proteins and carbohydrates, forming complexes that are difficult to digest, thus making them less accessible for absorption (Ojo, 2022). Fermentation decreased the initial oxalate present in kale by 50% (Figure 6). The lower pH attained during fermentation causes insoluble oxalate bound to calcium ions to solubilize. The soluble oxalate in the fermentation mixture is then utilized as an energy source by oxalotrophic bacteria such as *Lactobacillus acidophilus*, ultimately reducing the oxalate contents of the fermented kale (Wadamori et al., 2014; Weese et al., 2004). Fermentation decreased the phytate content but statistical significance was not reached. Phytases are enzymes produced by *lactobacilli* and other bacteria during fermentation and can break down and decrease phytate concentration in foods and increase the availability of minerals (Kim, 2017; Samtiya et al., 2020). Tannins occur in many plant‐based foods and beverages and have some antioxidant and antimicrobial properties but can also negatively impact nutrient absorption and digestion. Fermentation significantly decreased tannin content by up to 65% in kale; lactic acid bacteria produce tannases that break down tannins (Fahmia et al., 2019).

As expected, LPS treatment triggered the production of inflammatory cytokines iNOS, IL‐1β, TNF‐α, and IL‐6 in RAW 264.7 cells. Kale extracts, either fermented or unfermented, protected against this inflammation. When cells were treated with kale extract prior to stimulation by LPS, inflammation was prevented (Figure 8). No significant difference was observed between fermented and unfermented kale extracts. Polyphenols, quercetin and kaempferol, sulforaphane, indole‐3‐carbinol, and allyl isothiocyanate all contribute to confer the anti‐inflammatory properties of kale. These compounds interfere with the production of inflammatory substances like Nrf2 (nuclear factor erythroid 2‐related factor 2), which regulates the expression of various antioxidant and detoxifying enzymes in cells and regulates immune responses; suppresses the production of pro‐inflammatory cytokines (Tilg, 2015). It is likely that the compounds present in unfermented kale are sufficient to prevent inflammation. Thus although fermentation increases accessibility of these compounds, it does not confer any superior anti‐inflammation activity compared to the unfermented vegetable.

Compared to unfermented kale, fermentation resulted in a different surface microbiota composition of the vegetable. One key change was a reduction in taxa from the phylum Proteobacteria, particularly the orders Desulfovibrionales and Rhodobacterales (Figure 8C) in groups fermented by lactic acid bacteria. Cyanobacteria was also reduced by fermentation. Several species among Proteobacteria are often associated with disease and have been identified as a possible marker of microbiota instability, or dysbiosis, thus predisposing to disease (Moon et al., 2018). Proteobacteria and sulfate reducing Desulfovibrionales are a source of proinflammatory LPS. A decrease in Proteobacteria was not observed in kale fermented by natural fermentation or 2% salt. While lactic acid bacteria fermentation reduced Proteobacteria, it increased the representation of the genus *Lactobacillus*, particularly the species used as starter culture. *Lactobacillus* produces lactic acid that affects the taste, tanginess, and overall flavor of the fermented products. Thus, fermentation of kale may be a beneficial carrier for probiotic bacteria and further contribute to a lower representation of inflammatory promoting Proteobacteria.

In conclusion, while kale is established as ‘a functional food’, fermentation conferred a myriad of benefits that further enhanced this status. Specifically, fermentation increased the accessibility of polyphenols and sulforaphane while reducing the concentration of antinutritional factors oxalate and tannin. Furthermore, fermentation enhanced the antioxidant properties of kale and promoted its probiotic delivery potential while reducing the potential dietary delivery of inflammation promoting Proteobacteria. Fermentation with the combination of lactic acid bacteria (*L. acidophilus and L. lactis*) conferred the most benefits; fermentation with the natural method or 2% salt did not lower Proteobacteria surface bacteria and did not enhance the accessibility of total polyphenols and sulforaphane or enhance ABTS radical scavenging activity. The study has some limitations. While we show that fermentation reduced the concentration of β‐carotene and lutein in kale, we did not profile carotenoid derivatives that are formed by the action of bacterial enzymes and hence the significance of this finding is not clear.

## AUTHOR CONTRIBUTIONS

**Ujjwol Subedi:** Conceptualization (equal); investigation (equal); writing – original draft (equal). **Samnhita Raychaudhuri:** Investigation (equal); writing – review and editing (equal). **Si Fan:** Investigation (equal). **Opeyemi Ogedengbe:** Methodology (equal); writing – review and editing (equal). **Diana N. Obanda:** Conceptualization (equal); funding acquisition (equal); writing – review and editing (equal).

## FUNDING INFORMATION

The study was partially funded using the Faculty‐Student Research Award (FSRA) from the Graduate School, University of Maryland and partially funded by new laboratory startup funds awarded to Diana Obanda by the University of Maryland (UMD).

## CONFLICT OF INTEREST STATEMENT

The authors declare no conflict of interest.

## Data Availability

All data generated or analyzed during this study are included in this published article.
